# Hybrid gold/DNA nanowire circuit with sub-10 nm nanostructure arrays

**DOI:** 10.1038/s41378-020-00202-5

**Published:** 2020-11-02

**Authors:** Jong Seob Choi, Hye Bin Park, Jonathan H. Tsui, Byungyou Hong, Deok-Ho Kim, Hyung Jin Kim

**Affiliations:** 1 Department of Biomedical Engineering, Johns Hopkins University, Baltimore, MD 21205 USA; 2 Digital Healthcare Research Center, Gumi Electronics and Information Technology Research Institute (GERI), 350-27, Gumidaero, Gumi, Gyeongbuk, 39253 South Korea; 3 College of Information and Communication Engineering, Sungkyunkwan University, Suwon, 440-746 South Korea; 4 Department of Medicine, Johns Hopkins University School of Medicine, Baltimore, MD 21205 USA

**Keywords:** Nanowires, Electronic devices

## Abstract

We report on a simple and efficient method for the selective positioning of Au/DNA hybrid nanocircuits using a sequential combination of electron-beam lithography (EBL), plasma ashing, and a molecular patterning process. The nanostructures produced by the EBL and ashing process could be uniformly formed over a 12.6 in^2^ substrate with sub-10 nm patterning with good pattern fidelity. In addition, DNA molecules were immobilized on the selectively nanopatterned regions by alternating surface coating procedures of 3-(aminopropyl)triethoxysilane (APS) and diamond like carbon (DLC), followed by deposition of DNA molecules into a well-defined single DNA nanowire. These single DNA nanowires were used not only for fabricating Au/DNA hybrid nanowires by the conjugation of Au nanoparticles with DNA, but also for the formation of Au/DNA hybrid nanocircuits. These nanocircuits prepared from Au/DNA hybrid nanowires demonstrate conductivities of up to 4.3 × 10^5^ S/m in stable electrical performance. This selective and precise positioning method capable of controlling the size of nanostructures may find application in making sub-10 nm DNA wires and metal/DNA hybrid nanocircuits.

## Nanoscale Patterning with Precision

A fabrication method for manufacturing nanoscale circuits with high spatial resolution could provide a boost for high-sensitivity molecular detection devices. Electron-beam lithography (EBL) is a powerful tool for patterning nanowire circuits, but its performance declines at spatial scales of less than 10 nanometers. Researchers led by Dr. Deok-Ho Kim at the Johns Hopkins University and Dr. Hyung Jin Kim at the Gumi Electronics and information Technology Research Institute have overcome this limitation by coupling EBL with a plasma ashing procedure. Using this approach, they were able to precisely pattern nanostructures in a silicon substrate. They subsequently performed a series of chemical modifications that enabled them to couple DNA strands to these patterns, which in turn served as a template for gold nanowires. This strategy could accelerate the production of nanocircuits for biomolecular detection in diagnostics and other applications.

## Introduction

Since the discovery of chemically sensitive field effect transistors with Si nanowires^1^, the development of metal nanoscale electronic devices has been of growing interest^2–5^. 20 years later, the development of techniques to fabricate well-defined 1D metal nanowires at industrial scales is still intensively investigated worldwide due to the potential utility of such wires in electronic devices that require high degrees of sensitivity^6^. The sensitivity of 1D metal nanowires for biomolecular detection has been reported as higher than that of bulk metal detection because threshold voltage and surface charge effects increase with decreasing wire width^7^. However, the feasibility of utilizing 1D nanowires or nanowire arrays remains in question due to difficulties associated with mass production^8^. Moreover, precise positioning of these nanostructures is essential for enhancing their electrical sensitivity in electronic devices.

For fabricating nanowire patterns, electron-beam lithography (EBL) is one widely used top-down technology among diverse biomimetic nanopatterns technics^6,9^. Its high resolution, sub-20 nm feature sizes, and flexible range from nanoscale to microscale makes it possible to fabricate and utilize nano-gap electrodes, molecular arrays, and nanodevices. Hu et al. used poly(methylmethacrylate) resists to make sub-10 nm lines and well-aligned gold nanoparticle patterns on sub-10 nm lines^10^. Sun et al. also proposed a patterning method for sub-22 nm Si nanowire arrays using the EBL process^11^. Even though nanoscale features with high reproducibility were investigated by many researchers, the EBL process still suffers from a lack of reproducibility and low throughput of sub-10 nm features due to the prolonged exposure times required for large-area layouts.

Due to length controllability and binding affinity to various metal ions, alternative approaches for fabricating metal nanowires using DNA or protein assembly have been introduced^12,13^. These biomolecules can self-assemble via their charge interactions to make nanowires and can also hybridize with positively-charged metal ions or monomers. Moreover, due to its ease of experimentation and accessibility, it has emerged as a promising bottom-up approach to replace complex conventional photolithography processes. For example, Pate et al. have proposed a method of fabricating sub-10 nm Cu nanowires by using a solution-based linear DNA template^14^, and Watson et al. have reported on the mechanisms behind the formation of polymer nanowires on a linear DNA template^15^. Although DNA-templated metal nanowires are a new bottom-up approach and have been reported as a versatile template for fabrication of well-defined metal nanostructures^16–18^, the technical feasibility of producing 1D metal/DNA nanowire arrays with precise positioning of the nanowires is still not well established^19,20^.

Here, we describe a simple and efficient method to pattern sub-10 nm nanostructures with good pattern fidelity using EBL followed by a plasma ashing process. These methods enable selective positioning of sub-10 nm scale structures, which when combined with a selective molecular patterning process, can be used to fabricate Au/DNA hybrid nanowires and nanowire arrays. As Au nanoparticles have been one of the most widely used nanoparticles for biosensors^21^, these selectively-positioned Au/DNA hybrid nanowire arrays can potentially serve as an effective template for the fabrication of electrical biocircuits and devices.

## Results and discussions

**Fig. 1:**
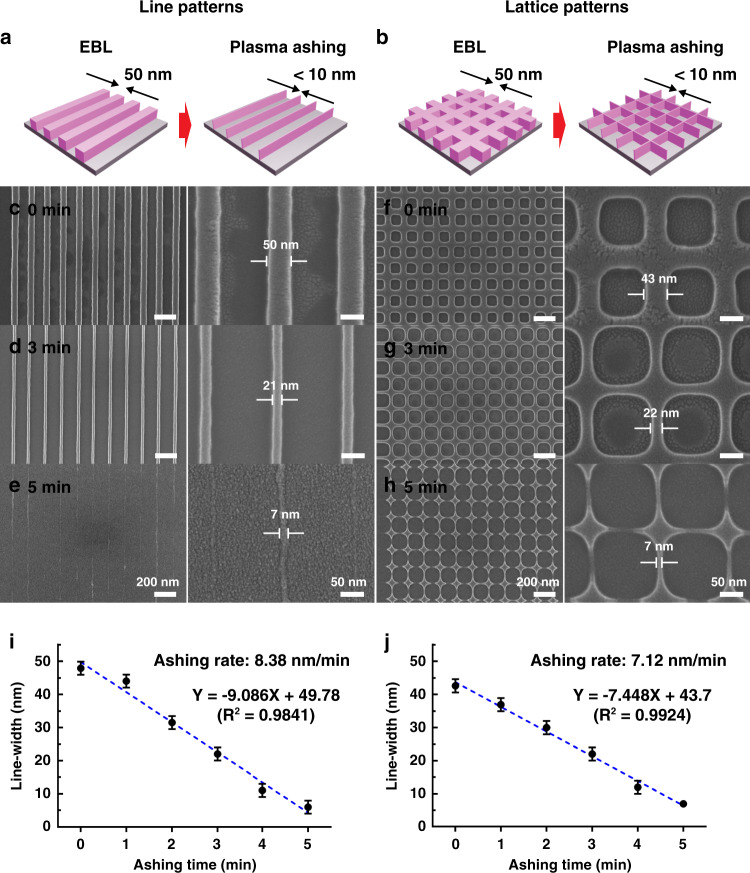
Generation of nanostructures with sequential EBL and ashing. Schematic illustration for generating sub-10 nm (**a**) line or (**b**) lattice patterns on the SiO_2_/Si wafer. Nanostructures with 50 nm-width were fabricated by electron-beam lithography (EBL) followed by plasma ashing treatment to reduce the width of nanopatterns down to sub-10 nm. **c**–**h** SEM images of nanolines and nanolattices with different width depending on varying plasma ashing time. Right panels are magnified images of left panels. (**i**) A plot of the line- and (**j**) lattice-width depending on the plasma ashing time. Plasma ashing process was conducted with time intervals of 1 min up to 5 min

Classical EBL technology can homogeneously make patterns with 50 nm feature sizes, but this method is not effective for producing sub-10 nm patterns. In this work, sub-10 nano line and lattice patterns were achieved by performing an additional ashing process following a conventional EBL process. Figure 1 provides a schematic illustration of the fabrication process and results of fabricating sub-10 nm structures produced by two successive EBL and plasma ashing processes on a SiO_2_/Si substrate. Initial line- or lattice-patterns with 50 nm widths were prepared by EBL. The dimensions of these structures were proportionally decreased as plasma ashing treatment time increased (Fig. 1c–h). A final 7 nm width pattern could be achieved with 5 min of plasma ashing treatment. The ashing rate in line patterns was about 8.38 nm/min and 7.12 nm/min for lattice patterns (Fig. 1i, j). The small difference in ashing rates on both line- and lattice patterns is due to the turbulent flow of plasma. Plasma flow in lattice patterns could be undergoing more irregular fluctuations than in line patterns due to the more complex nanostructures, thereby resulting in a slower ashing rate.

**Fig. 2:**
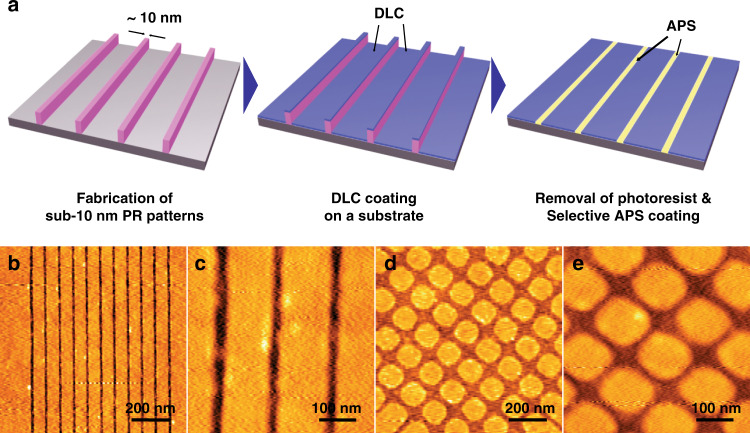
Selective deposition of DLC and APS to form nanowires. **a** Schematic illustration for selectively aligned DLC area by using surface-patterning techniques on the nanostructures. **b**, **d** AFM images representing line- and lattice-patterns after DLC coating treatment. **c**, **e** Higher magnification images of (**b**) and (**d**), respectively

**Fig. 3:**
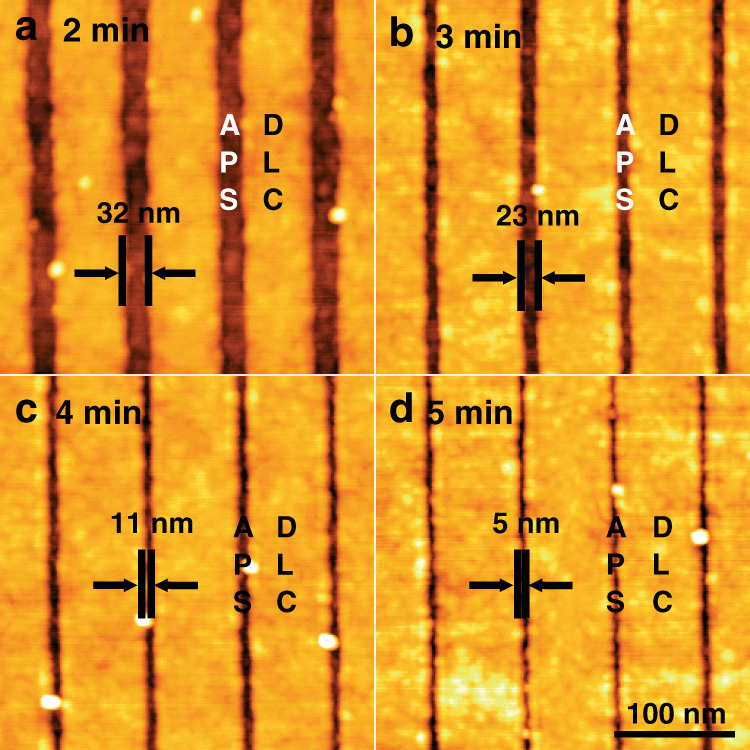
Representative AFM images for line-patterns composing the alternate APS and DLC layers on a substrate after different plasma ashing times. **a** 2 min: 32 ± 2 nm, (**b**) 3 min: 23 ± 2 nm, (**c**) 4 min: 11 ± 2 nm, (**d**) 5 min: 5 ± 2 nm

In order to create the selective positioning of DNA molecular patterns, 3-(aminopropyl)triethoxysilane (APS) and diamond like carbon (DLC) were used as an adhesion layer and passivation layer, respectively (Fig. 2a). First, sub-10 nm nanostructures were prepared by consecutive EBL and plasma ashing processes as illustrated in Fig. 1. Then, a DLC layer was coated on the nanopatterned substrate by plasma enhanced chemical vapor deposition (PECVD) to form a molecular blocking layer. DLC and APS-coated layers could be confirmed by AFM topography measurements after removing the photoresist nanostructures (Fig. 2b–e). Selectively-deposited DLC layers could be observed, and these layers can be used as a passivation layer since APS molecules do not attach to DLC molecules and can only bond to nanostructures (line or lattice patterns) as initially positioned. Representative AFM images of alternating APS and DLC molecular line patterns for 4 samples prepared with different plasma ashing times show that well-aligned molecular line patterns 32 ± 2 nm, 23 ± 2 nm, 11 ± 2 nm, and 5 ± 2 nm wide could be obtained after 2, 3, 4, and 5 min of ashing, respectively (Fig. 3).

**Fig. 4:**
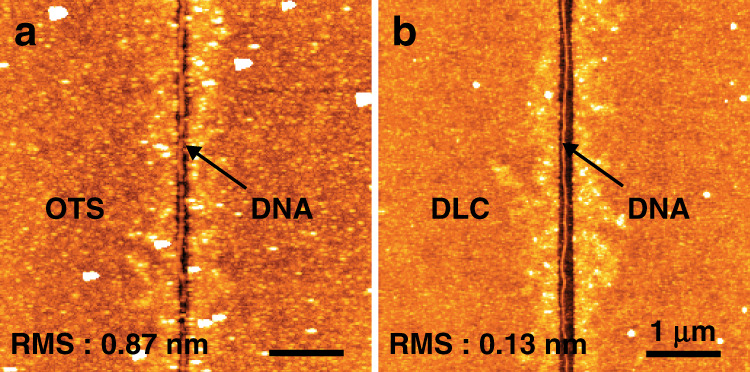
A comparison of OTS and DLC as passivation layers for the formation of DNA nanowires. **a** Schematic illustration for selective alignment of DNA molecules on a substrate. DNA wires on (**b**) APS/OTS passivation layer, and (**c**) APS/DLS passivation layer

APS and DLC layers play key roles in the selective attachment of DNA through strong Coulombic interactions between the -NH_3_^+^ groups of APS and the negatively-charged phosphate backbone of DNA, and weak interactions between the hydrophobic DLC and DNA. This selective immobilization of DNA molecules along APS-coated regions can facilitate DNA nanowire assembly (Fig. 4). Prior studies had difficulty controlling the selective positioning of immobilized single DNA nanowires, and it remains a challenging issue^20^. However, EBL followed by plasma ashing and combined with a molecular surface patterning method can achieve precise positioning of single DNA nanowires. Prior to forming single DNA nanowire arrays on the APS coated region, we compared octadecyltrichlorosilane (OTS) to DLC in formation of single DNA nanowires as OTS is also known as a passivation coating reagent^22,23^. In our previous study, contact angle values for OTS-, DLC- and APS-coated surface were 95°, 75° and 55°, respectively^24^. Therefore, a DLC layer possesses hydrophobicity similar to an OTS layer. After OTS treatment of the substrate, the average RMS value of surface roughness was 0.87 nm, which is higher than that of substrates coated with DLC (0.13 nm). The relatively rough surface of OTS might interrupt and confine some DNA and water molecules from moving, which leads to DNA fragments remaining on the OTS surface (Fig. 4a). On the other hand, the smoother surface of DLC appears to be more effective at preventing non-specific attachment of DNA molecules. Most of the DNA and water molecules tended to move to the hydrophilic APS regions while the solution was evaporated or removed over time, resulting in better formation of single DNA nanowires (Fig. 4b).

**Fig. 5:**
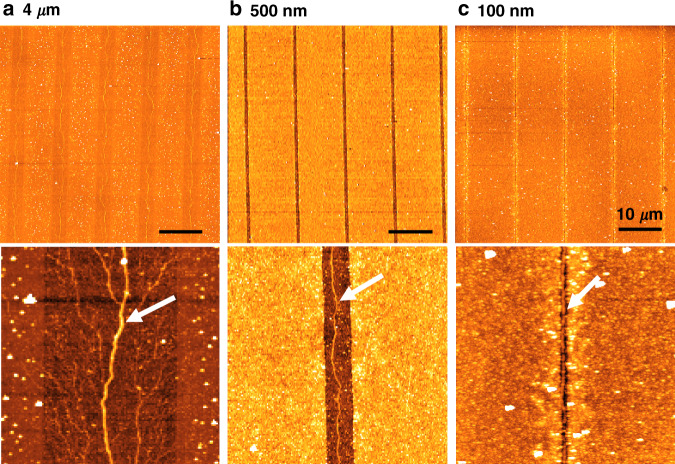
Nanopattern line width influences the alignment of DNA nanowires. (top) DNA alignment on the APS regions. Bottom images are magnified from top images. White arrows indicate the DNA nanowire on the APS region. **a** 4 μm, (**b**) 500 nm, (**c**) 100 nm. Bottom image in (**c**) is the same with Fig. 4 (**b**)

To investigate the effects of the nanopattern width on DNA nanowire alignment, various widths of nanopatterns with 4 μm, 500 nm, and 100 nm were fabricated. It was found that line width after removal of the nanostructures is one of the critical parameters for achieving good DNA nanowire alignment. When the line width was relatively wide (Fig. 5a), the generated DNA nanowire formed as branched lines, which would result in branched Au nanowires. However, as line widths became narrower, the immobilized DNA nanowires became single lines as the reduced deposition area helped to concentrate the DNA molecules and prevent spreading. For 500 nm-wide lines, a single DNA nanowire could be formed, albeit in a curved shape (Fig. 5b), whereas for 100 nm-wide lines, a straight DNA nanowire along the direction of the nanopatterned line was formed (Fig. 5c).

**Fig. 6:**
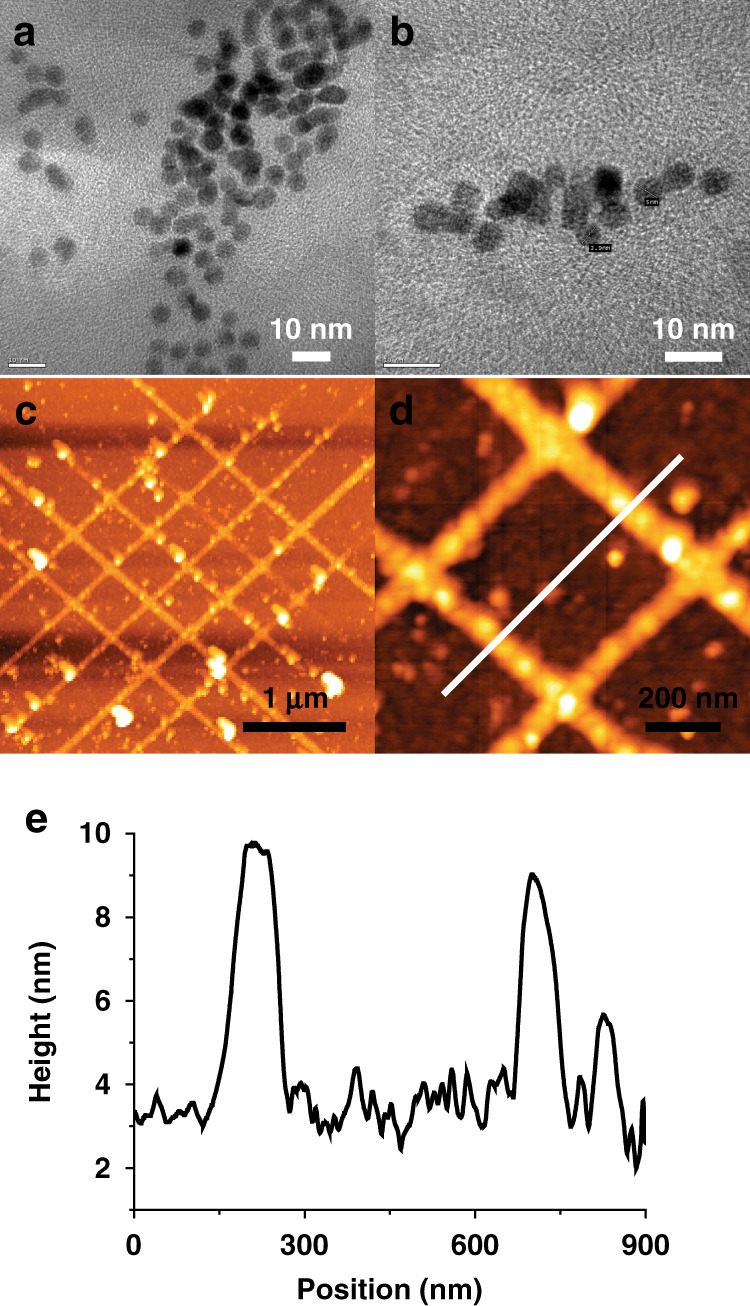
Fabrication of DNA-Au hybrid nanowires. **a**, **b** Representative TEM images of Au nanoparticles used for assembly of Au nanowires. **c**, **d** AFM topography for DNA-Au hybrid nanowires aligned on the SiO_2_/Si substrate. **e** A height profile represented by the white line in (**d**)

The bonding mechanism of positively charged Au nanoparticles to negatively charged DNA molecules has been previously reported^16^. A variety of metal ions such as Ag, Au, Pt, Cu, Ni, Fe, Co, and Pd react with the phosphate groups in DNA by electrostatic interaction, resulting in seed nuclei for the growth of metal nanoparticles. However, precise positioning of a two-dimensional single metal nanowire network is still a challenging issue^23,25^. Here we have demonstrated the fabrication of sub-10 nm nanostructures on a 4-inch diameter wafer by using EBL and plasma ashing processes followed by alternating surface patterning methods for selectively located Au/DNA hybrid nanowire arrays. In total, 5 nm Au nanoparticles (Fig. 6a, b) were deposited onto DNA nanowires in lattice patterns (Fig. 6c, d). The average height of produced DNA-template Au nanowires was 7 nm and the average width was ~60 nm (Fig. 6e). We also attempted to reduce the width of DNA-template Au nanowires to fit sub-10 nm initial nanostructures, but the Au nanoparticles did not properly assemble on these structures due to nanoparticle aggregation.

**Fig. 7:**
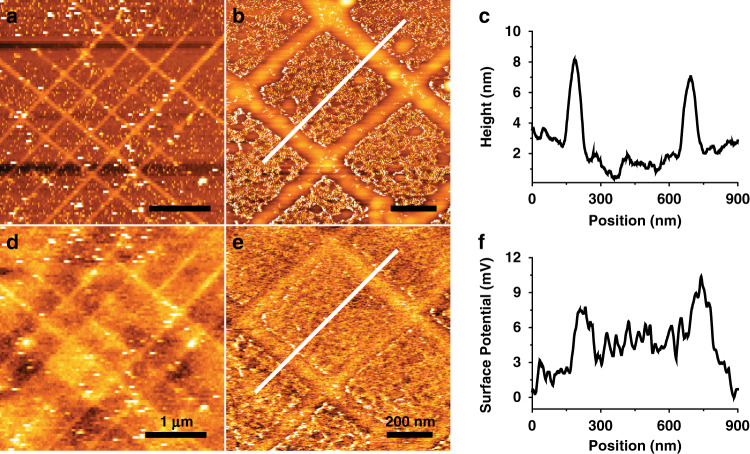
KFM study for high resolution surface potential and topography mapping. **a**, **b** KFM topography mapping of hybrid Au/DNA Nanowire array. **c** A height profile of the white line in (**b**). **d**, **e** KFM surface potential images. **f** A surface potential profile represented by the white line in (**e**)

In most cases, this process enables the linear alignment and deposition of Au nanoparticles or clusters onto single DNA nanowires; however, if there is the presence of a nonmetallized portion or if there is no connection between the Au particles in the nanowire, the conductivity of the Au/DNA hybrid nanowire or network would be negatively impacted. To measure the degree of conductivity of Au/DNA nanowires and to check their integrity, a Kelvin probe force microscope (KFM) study was carried out to obtain surface potential values. Like with the topographical measurements obtained with AFM, KFM analysis also showed that the crosshatched areas of the Au nanowire lattices were better assembled and appeared more distinctly than the line patterns (Fig. 7a, d). It is likely that this is due to a higher positive charge density at the crosshatch area compared to that along individual lines, resulting in a large amount of negatively-charged DNA molecules being immobilized on the crosshatched sections. As a result, most of the positively-charged Au nanoparticles were then preferentially localized to the greater number of DNA molecules in these areas. Measured maximum surface potential was ~6 mV along the Au/DNA nanowire. The measurement of surface potential of the nanowire-based sensors is a crucial parameter for understanding and predicting the performance of nanowire sensors^26^, as reacting metal nanowires with biomolecules via charge interactions can result in a subsequent alteration of nanowire conductivity.

**Fig. 8:**
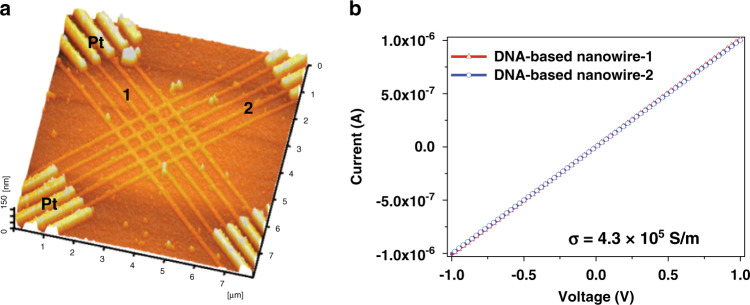
Nanocircuit fabrication using Au/DNA hybrid nanowire arrays fixed between two Pt electrodes. **a** AFM topography image with the numbers 1 and 2 indicating each nanowire line array. **b** Electrical properties (current-voltage) of nanocircuits made from Au/DNA hybrid nanowires

To further assess the electrical properties of fabricated hybrid Au/DNA nanowires, two Pt electrodes were fabricated at the ends of nanowire arrays (Fig. 8a). The linear relationship between current and voltage shows an ohmic behavior, an indication that Au/DNA hybrid nanowires possess metallic properties (Fig. 8b). The conductivity of Au/DNA hybrid nanowires was about 4.3 × 10^5^ S/m, which is 2 orders of magnitude lower than pure Au electrodes^27^. In addition, there was no difference in conductivity between the two sets of nanowire arrays (1, 2) shown in Fig. 8a. This is likely due to there being no nonmetallized portions or connection breaks between Au nanoparticles in the nanowires. The reported conductivity of linear Au/DNA nanowires fabricated using a similar method (3.2 × 104 S/m) was lower than that of the grid patterns of hybrid nanowires fabricated in this study^28^. This is likely due to the high concentration of DNA molecules at the grid intersections that would result in a greater density of Au nanoparticles that are present than there would be in a linear Au/DNA nanowire. Moreover, electrode sensitivity is greatly related to the device quality such as electrical conductivity and impedance^29^. Surface area and charge potentials at the surface are one of the important parameters to enhance the electrode performance in recording sensitivity. Surface area of grid patterns is higher than that of line patterns. We believe that this is a result of a relatively high accumulation of potentials at the surface areas of the grid Au/DNA patterns that can then cause a high density of Au nanoparticles to be attached to the biomolecules.

Overall, uniformly formed sub-10 nm structures over a 4-inch wafer-size with good pattern fidelity was achieved by using a combination of EBL and plasma ashing. By an additional surface patterning process, selectively-aligned and positioned hybrid Au/DNA nanowires and arrays could be fabricated onto sub-10 nm structures. Our approach to fabricating Au/DNA nanocircuits is a promising technique for nanoelectronics applications that require precise assembly and positioning of electrodes and wiring. These Au/DNA hybrid nanocircuits could be further developed and applied as biomedical and environmental sensors through integration with appropriate technologies such as nano/microfluidics and molecular biology for early-detection diagnostics^17,30^.

## Methods

Aligned hybrid DNA/Au nanowires were produced as follows: (1) A ma-N2401 negative tone electron beam resist (MicroResist Technology) was coated on top of a silicon substrate with a 200 nm-thick oxide layer (WaferWorks Corp.). The thickness of the resist was held at ~100 nm by optimizing the spin-coating speed (1000 rpm for 120 s). In order to form nanopatterns with uniform intervals, the resist was exposed to electron beam light (JEOL, JBX6000FS) with a dose of 200 µC/cm^2^ at 25 kV. The exposed layer was developed with AZ726 (MicroChemicals) for 80 s and then rinsed with double distilled water for 60 s. As a result, 50 nm width nanopatterns were produced with intervals of 100 nm. (2) The nanopatterned structures were ashed by using a chemical dry etcher (Tokuda Co., CDE-7-3) with O_2_ (1500 sccm), N_2_ (500 sccm), and CF_4_ (100 sccm) at 50 W microwave power (2.45 GHz). (3) Then, a DLC thin film with a thickness of 5 nm was deposited on the nano-patterned substrate by RF PECVD with methane (CH_4_) and hydrogen (H_2_) gas at 150 W RF power (13.56 MHz). (4) After lift-off of the nanopatterned structures, the substrate was immersed in a 0.1 mM 3-aminopropyltriethoxysilane (Sigma-Aldrich) solution diluted in anhydrous toluene (Sigma-Aldrich) for 30 min. (5) A λ-DNA (Bio Basic Inc., 48,502 bps) solution of 30 mL was prepared in a concentration of 10 ng/mL by TE buffer (10 mM tris-HCl and 1 mM EDTA, pH 8.0; Noble Biosciences. Inc.) and deposited on APS regions and allowed to react for 3 min. (6) DNA molecules were treated with an aniline (Sigma-Aldrich)-capped gold nanoparticle (AN-AuNP) solution of 60 µL to form hybrid nanowires and, after 30 min, the samples were rinsed with double distilled water. The samples were observed with an atomic force microscope (AFM, SII Nanotechnology, SPA 400) and field emission-scanning electron microscope (FE-SEM, JSM-7610F).

In order to measure electrical properties of Au/DNA nanowire arrays, titanium/platinum (Ti/Pt) electrodes (250 nm in width, 250 nm in space between Ti/Pt electrodes) on each end of Au/DNA chain was fabricated by conventional photolithography. Then, thermal evaporation was conducted to sequentially deposit 50 nm of Ti and 150 nm of Pt on the entire wafer. After removing the photoresist underneath Ti/Pt layer, Ti/Pt electrodes were selectively positioned and connected with Au/DNA nanowires. The conducting property was characterized using a precise semiconductor parameter analyzer (SnM-ICVMS100).
